# Association between patellar tendon-related pain and morphological abnormalities of the patellar tendon visualized using ultrasound imaging in middle school athletes

**DOI:** 10.1371/journal.pone.0357407

**Published:** 2026-09-08

**Authors:** Yuuka Yasuura, Takahiko Fukumoto, Hiroaki Yamano, Yuya Mawarikado, Daisuke Uritani

**Affiliations:** 1 Graduate School of Health Sciences, Kio University, Nara, Japan; 2 Department of Rehabilitation, Shimada Hospital, Osaka, Japan; 3 Graduate School of Medicine, Musculoskeletal Reconstructive Surgery, Nara Medical University, Nara, Japan; Universiti Malaya, MALAYSIA

## Abstract

**Background/Objective:**

To investigate whether morphological abnormalities of the patellar tendon, assessed using ultrasound imaging, are associated with patellar tendon-related pain in adolescent soccer players.

**Methods:**

This cross-sectional observational study was conducted at a university and enrolled 140 middle school soccer players (280 knees). Pain assessment was conducted using the visual analog scale and tenderness. Physical function assessments were also conducted. Ultrasound imaging was used to evaluate the morphology of the patellar tendon.

**Results:**

Data on a total of 211 knees were analyzed. Compared with non-painful knees, knees with patellar tendon-related pain exhibited significantly lower knee extension strength and a higher prevalence of hypoechoic and Doppler findings. The logistic regression analysis demonstrated a significant association between hypoechoic findings and patellar tendon-related pain (adjusted odds ratio = 10.65, 95% confidence interval: 3.65–31.88, p < 0.001), whereas no significant associations were observed for other morphological or physical function measures.

**Conclusion:**

Hypoechoic findings in the patellar tendon were significantly associated with patellar tendon-related pain in adolescent soccer players. These findings suggest that ultrasound imaging may be useful for identifying tendon characteristics that are associated with pain. However, longitudinal studies are needed to establish causal relationships.

## Introduction

The prevalence of sports injuries among adolescent athletes ranges from 15% to 32% [1,2], with knee joint injuries being particularly common. Patellar tendon-related pain is a symptom observed in conditions such as Osgood–Schlatter disease (OSD) and jumper’s knee, which are representative knee disorders that frequently occur during growth. These conditions increase in prevalence up to the age of 18 years [3]. Approximately 40% of adolescents who experience knee pain continue to report it a year later, indicating that such pain does not necessarily have a favorable prognosis [4]. These disorders can potentially affect the athletes’ ability to continue participating in sports, making early detection and preventive measures crucial [5].

Patellar tendon-related disorders, such as OSD and jumper’s knee, are associated with physical function [6]. These disorders are frequently reported to be associated with decreased flexibility of the quadriceps and hamstrings [6–9] as well as other factors, such as reduced ankle dorsiflexion range of motion [10], increased knee extension strength [6], and decreased hip extension strength [11]. However, in clinical practice, patellar tendon-related pain often occurs even in the absence of notable flexibility and strength deficits.

Patellar tendon-related disorders result in increased proteoglycan substrate levels and neovascularization, leading to edema and disruption of collagen arrangement in the patellar tendon tissue. Ultrasound imaging (US) is a noninvasive, cost-effective, and widely used modality that enables real-time evaluation of patellar tendon morphology, including thickening, hypoechoic areas, and neovascularization, with improved resolution compared with magnetic resonance imaging (MRI) [12,13]. Morphological abnormalities of the patellar tendon observed on US, such as thickening, hypoechoic findings, and neovascularization, are thought to reflect tissue remodeling, inflammation, and microstructural damage [14,15] and have been suggested as potential risk factors for the development of painful patellar tendinopathy [16]. As adolescence is a period of rapid tendon growth and adaptation [17], morphological abnormalities occurring during this phase may have important clinical implications. However, despite growing interest in tendon morphology, limited research has focused on middle school athletes undergoing active growth, and the relationship between morphological abnormalities and physical function in this population remains unclear. In this study, we aimed to investigate whether morphological abnormalities of the patellar tendon observed on US are associated with patellar tendon-related pain, compared with measures from other physical function evaluations. We hypothesized that morphological abnormalities of the patellar tendon, particularly hypoechoic findings, would be more strongly associated with patellar tendon-related pain, compared with measures of physical function.

## Materials and methods

### Participants

This cross-sectional observational study used a convenience sample. Participants were recruited between March 30, 2023 and June 30, 2023. During this period, we enrolled 140 male middle school students affiliated with a soccer team, resulting in a cohort of 280 knees. Individuals with substantial lower limb deformities, history of lower limb surgery, or neurological symptoms affecting the lower extremities were excluded from the study cohort.

### Experimental preparation

In a preliminary questionnaire, participants were asked to provide information regarding their height, weight, age, sex, dominant foot, medical history, and years of soccer experience. The dominant foot was defined as the foot used to kick the ball.

#### Pain assessment.

The painful knee was defined as a knee with tenderness on palpation of the patellar tendon and self-reported pain (visual analog scale score > 0) at rest (seated position) or during sports activity [18,19]. A single physical therapist assessed tenderness, and a knee was considered tenderness-positive when pain was elicited upon palpation of the tendon substance or its insertion.

#### Physical function assessment.

Physical functions were measured using straight leg raising (SLR) [20], ankle dorsiflexion range of motion (ROM), heel-buttock distance (HBD) [21], knee extension strength, knee flexion strength, and hip extension strength.

SLR passive ROM was measured in 5° increments using a goniometer. The SLR test was performed with the participants in the supine position, extending their knee fully while flexing their hip. The intra-rater reliabilities of this assessment are well-established [20]. Ankle dorsiflexion ROM was measured using a gravity inclinometer [22]. The lines were placed on the floor and wall at right angles to each other (referred to as the floor line and wall line, respectively). The participants positioned their foot so that the middle of their heel and second toe were aligned with the floor line. Keeping their knee aligned with the floor line, the participants tilted their tibia forward to touch the center of their patella toward the wall line. At the point of maximum involvement, a gravity inclinometer was placed on the anterior border of the tibia (15 cm below the tibial tuberosity), and the angle relative to the vertical was recorded. The intra- and inter-rater reliabilities of this assessment are well-established [22,23].

Tightness of the quadriceps femoris was assessed by measuring HBD with the participant in the prone position with the knee maximally flexed [7,21]. Knee extension and flexion [24] and hip extension muscle strength [25] were measured using a handheld dynamometer (μTas F-1, Anima Inc, Tokyo, Japan). This measurement technique was based on the findings of a recent systematic review that concluded that the dynamometer was a reliable and valid instrument for assessing muscle strength in clinical settings [23], [26]. Maximum isometric contraction was performed for 5 s. The measurements were conducted exclusively by physical therapists with 5–10 years of experience. The intra- and inter-rater reliabilities of the measurement of knee extension, knee flexion, and hip extension using a handheld dynamometer are established [24,25].

### US evaluation

Patellar tendon thickening, hypoechoic areas, and neovascularization were observed using a high-frequency linear probe (L11–3; 3–11 MHz) attached to an ultrasound system (SONIMAGE MX1; Konica Minolta, Inc., Tokyo, Japan). The imaging depth was fixed at 3.5 cm for all participants, and gain settings were adjusted by the examiner at the beginning of each examination to ensure adequate visualization of the tendon. Patellar tendon thickening was measured by observing the patellar tendon in the longitudinal plane and measuring its thickness at a point 5 mm distal to the inferior pole of the patella [14]. The hypoechoic areas of the patellar tendon were assessed in the longitudinal direction by observing the area from the attachment site at the patella to the tibial tuberosity. Neovascularization was assessed using the color Doppler method. The measurements were conducted exclusively by a physical therapist with 5 years of experience in US.

### Data processing

Muscle strength was evaluated twice, and the highest value was recorded and normalized to the body weight. Tendon thickness was defined as the distance between its superficial and deep layers [27], and it was measured using ImageJ (version 1.54) [14]. In accordance with a previous study, if a hypoechoic area measuring ≥ 2 mm in length and width was observed in the patellar tendon, it was considered hypoechoic-positive (+) [28]. Neovascularization was assessed by observing vascular structures longer than 1 mm in the sagittal plane of the tendon, classified as positive (+) for Doppler [14]. The image analysis process demonstrated high intra-rater reliability [14]. To minimize bias, the analyst was not informed of whether each knee was painful or not during the image evaluation.

### Statistical analysis

The normality of data was assessed using the Shapiro–Wilk test. As the data were non-normally distributed, the Mann–Whitney *U* test was used to compare knees with and without patellar tendon-related pain. The proportions of hypoechoic- and Doppler-positive findings were compared using the chi-square test. Variables with a p-value < 0.05 in the initial analysis were included as independent variables, and the presence or absence of pain was set as the dependent variable in the logistic regression analysis using the forced entry method. The goodness of fit of the logistic regression model was assessed using the Hosmer–Lemeshow test. To address the potential non-independence of bilateral knees, a sensitivity analysis at the participant level was performed using one knee per participant; the painful knee was prioritized when present. However, for knee extension strength, the mean value of both knees was used to provide a single representative value for each participant. The same statistical procedures were applied as in the primary analysis. All statistical analyses were performed using SPSS (version 22.0; SPSS Inc., Chicago, IL, USA), with a significance level set at < 5%.

### Ethics statements

This study was conducted in accordance with the Declaration of Helsinki. The Research Ethics Committee of Kio University (reference number: R4-48) approved this study, and all participants and their parents provided written informed consent before participation in this study. The authors did not have access to identifiable personal information during or after data collection.

## Results

**Fig 1:**
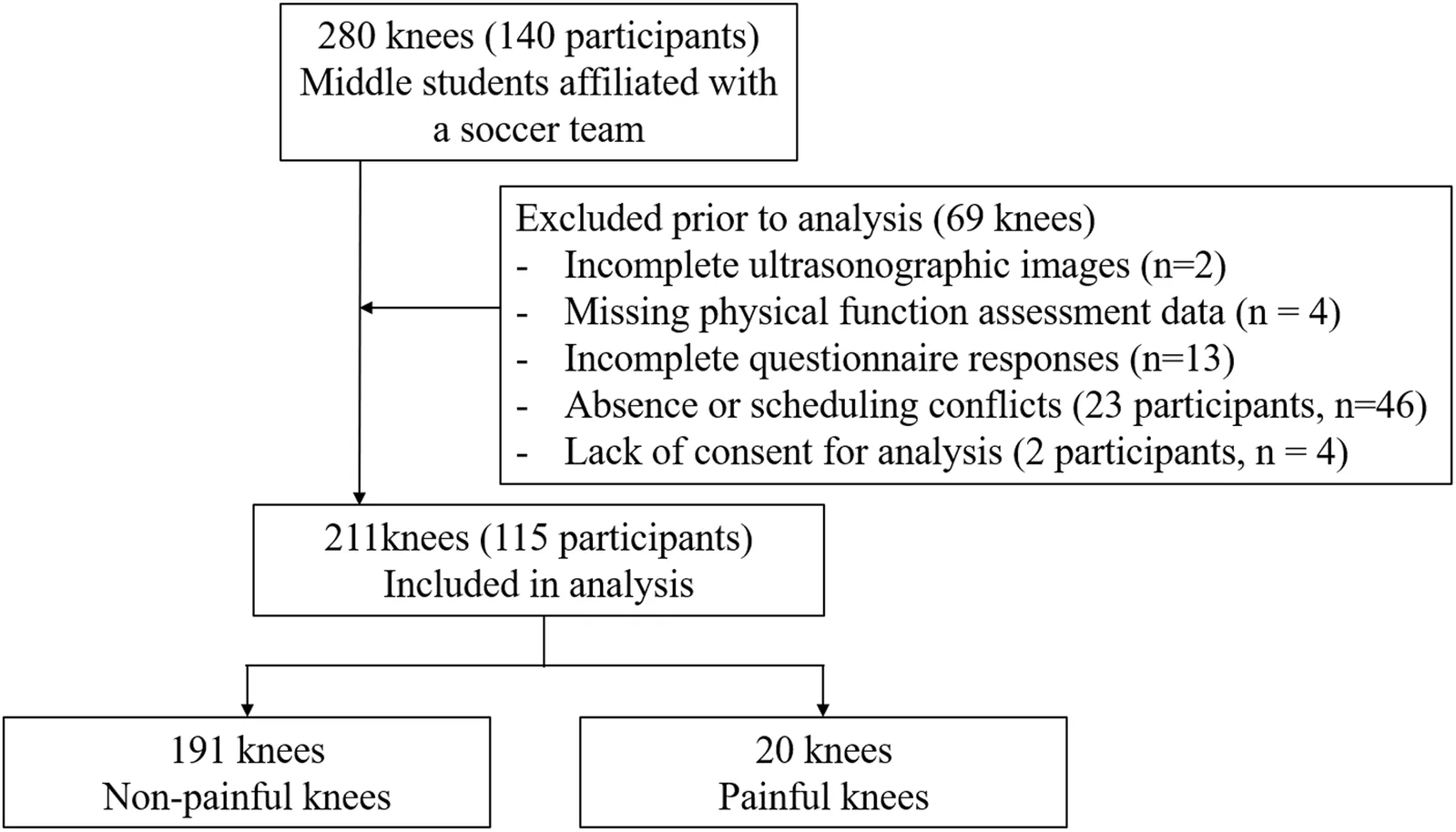
Study flow diagram.

Of the 280 knees (140 participants) which were initially assessed, 211 knees (115 participants) met the inclusion criteria and their data were included in the final analysis after applying the exclusion criteria. The demographic characteristics of the participants are presented in Table 1. The remaining knees were categorized thus: 191 knees without pain (non-painful knees) and 20 knees with tenderness in the patellar tendon (painful knees) (Fig 1). Mann–Whitney *U* tests revealed significant differences in knee extension strength between the painful and non-painful knees. Additionally, chi-square tests showed significant differences in the proportions of hypoechoic- and Doppler-positive findings between painful and non-painful knees (Table 2). The logistic regression analysis revealed a significant association between patellar tendon-related pain and hypoechoic-positive finding (Table 3). The Hosmer–Lemeshow goodness-of-fit test indicated that the model fit was acceptable (p = 0.148).

**Table 1:** Demographic characteristics of the participants (N = 115).

Variable	Value
**Age**	12.9 ± 0.8
**BMI,**^**a**^ **kg/m**^**2**^	18.5 ± 2.1
**Dominant side (right/left)**	104/11

^a^BMI: body mass index

**Table 2:** Comparison of outcomes between painful and non-painful knees.

	Painful knees (20 knees)	Non-painful knees (191 knees)	p-value^*^
SLR,^°a, c^	50.0 (30.0–70.0)	65.0 (35.0–75.0)	0.52
Ankle dorsiflexion ROM,^°a, d^	47.4 (41.3–50.0)	45.0 (40.0–49.5)	0.77
HBD, cm^a, e^	0.0 (0.0–10.0)	0.0 (0.0–2.8)	0.16
Knee extension strength, kgf/kg^a^	0.6 (0.6–0.7)	0.8 (0.6–0.8)	0.03
Knee flexion strength, kgf/kg^a^	0.3 (0.3–0.4)	0.4 (0.3–0.4)	0.89
Hip extension strength, kgf/kg^a^	0.5 (0.5–0.6)	0.6 (0.4–0.6)	0.66
Thickness, cm^a^	3.2 (2.7–3.3)	3.4 (2.9–3.7)	0.31
Hypoechoic-positive finding: n^b^	13 (65)	21 (11)	< 0.001
Doppler-positive finding: n^b^	10 (50)	33 (17)	< 0.001

^a^: Analyzed using the Mann–Whitney *U* test. Values are presented as median (interquartile range: 25–75%).

^b^: Analyzed using the chi-square test. Values are presented as n (percentage).

^c^SLR: straight leg raising

^d^ROM: range of motion

^e^HBD: heel-buttock distance

*Statistical significance at p < 0.05

**Table 3:** Results of the logistic regression analysis of the factors associated with patellar tendon-related pain (n = 211 knees).

Variable	B^a^	Exp(B)^b^	95% CI^c^ (minimum to maximum)	p-value^*^
**Knee extension strength**	−3.34	0.04	0.00–1.90	0.08
**Hypoechoic-positive finding**	2.37	10.65	3.65–31.88	< 0.001
**Doppler-positive finding**	1.01	2.75	0.92–8.20	0.07

^a^B: logistic regression coefficient

^b^Exp(B): odds ratio

^c^95% CI: 95% confidence interval

*Statistical significance at p < 0.05

Sensitivity analysis at the participant level was performed using one observation per participant. For knee extension strength, the mean value of both knees was used for each participant, and group comparisons were performed using the Mann–Whitney U, chi-square, or Fisher’s exact test, as appropriate. The results were consistent with those of the primary analysis: hypoechoic and Doppler-positive findings remained significantly more frequent in participants with pain than in participants without pain, whereas knee extension strength did not differ significantly between the groups (S1 Table). These findings were consistent with those in the primary knee-level analysis, supporting the robustness of the association between hypoechoic findings and patellar tendon-related pain.

## Discussion

### Main findings

The findings of the present study support our hypothesis, as patellar tendon-related pain was significantly associated with hypoechoic findings. Furthermore, the multivariable logistic regression analysis demonstrated that hypoechoic findings in the patellar tendon were significantly associated with pain, whereas physical function measures showed weaker or no significant associations.

The participant-level sensitivity analysis yielded results that were largely consistent with those in the primary knee-level analysis, particularly regarding the significant association between hypoechoic findings and pain. Although Doppler findings reached statistical significance in the sensitivity analysis, this was not observed in the multivariable model, suggesting that its association with pain may be less robust and may have been influenced by the analytical approach or the limited sample size. Therefore, these findings should be interpreted cautiously, as the participant-level analysis was intended to support, not replace, the primary knee-level analysis.

### Prevalence rate of patellar tendon-related pain

The prevalence rate of OSD is reported to be between 9.8% and 16% [6,10,29], whereas that of jumper’s knee ranges from 8.5% to 14.2% [30,31]. In the present study, the prevalence of patellar tendon-related pain disorders was 9.4%. Although no formal diagnosis was made, the prevalence observed in this study was comparable to those reported in previous studies.

### Relationship between patellar tendon-related pain and morphological abnormalities of the patellar tendon

Hypoechoic findings commonly reflect inflammation-related changes, such as edema or fluid accumulation and are often associated with pain [17,32]. Reportedly, soccer players impose a high mechanical load on the patellar tendon due to the sport’s substantial demands on leg extensor velocity and power [33]. In the adolescent soccer players included in this study, prolonged exposure to mechanical stress contributed to microdamage in the patellar tendon, leading to increased proteoglycan production, collagen separation, and angiogenesis [34,35]. The proliferation of proteoglycans increases the water content within the tendon [33], which is visualized as a hypoechoic finding on US. Structural damage to the tendon has been demonstrated to be correlated with pain severity, with a higher likelihood of increased pain intensity [17]. These findings suggest that the association between a hypoechoic finding and patellar tendon-related pain in this study may be attributed to these pathological changes.

Doppler findings are considered to reflect neovascularization and have been suggested to be associated with pain in tendon disorders [36,37]. In contrast, other studies have reported that Doppler findings do not necessarily indicate tendon-related pain [38]. Similarly, in this study, we did not identify a significant association between Doppler-detected neovascularization and patellar tendon pain. Previous research has indicated that Doppler signals may persist even after pain resolution and can be observed for as long as 3–4 years following the onset of patellar tendinopathy [36]. Doppler findings suggest that neovascularization alone may not be sufficient to elicit pain and that other factors, such as neural ingrowth [39] and biochemical mediators [40], may contribute to symptom manifestation. Therefore, Doppler US findings should be interpreted with caution and in conjunction with clinical symptoms when assessing tendon pain. Recent evidence further supports the multifactorial nature of patellar tendon-related pain. Tayfur et al. [41,42] reported that, in addition to structural changes, psychosocial and clinical factors are significantly associated with patellar tendinopathy in jumping athletes, suggesting that ultrasonographic abnormalities represent only one aspect of the pain experience. Moreover, a recent systematic review and meta-analysis demonstrated that altered landing mechanics and impaired neuromuscular control are also associated with patellar tendinopathy. Together, these findings indicate that tendon-related pain arises from the interaction of structural, psychosocial, biomechanical, and neuromuscular factors, and they cannot be fully explained by imaging findings alone. Previous studies have reported that repetitive overload induces a non-inflammatory proliferative response, resulting in uniform thickening of the tendon and an increase in its cross-sectional area, which helps alleviate the stress (force per unit area) applied to the tendon [34,35]. The thickness of the patellar tendon itself is not necessarily linked to pain; rather, it may be a physiological response to maintaining tendon strength [43]. Additionally, in adult athletes, it has been reported that a patellar tendon thickness ≥ 4 mm at the proximal insertion site can be interpreted as an early sign of degeneration [40]. In the present study, the mean patellar tendon thickness in the pain and no-pain groups was < 4 mm. These findings suggest that, although excessive thickening of the patellar tendon may be associated with pain beyond a certain threshold, hypertrophy itself is a physiological response to maintaining tendon strength, which may explain why no association with pain was observed in this study. However, data on the relationship between patellar tendon hypertrophy and pain in middle school students remain limited; this subject therefore warrants further investigation.

### Limitations

This study has certain limitations. The sample size was small, which may limit the generalizability of the findings. Additionally, data from 69 knees were excluded owing to missing measurements or lack of consent, as illustrated in the study flowchart. Although missing data were primarily related to logistical or consent-related issues, the possibility that the missingness was not completely random cannot be excluded, and this may have introduced selection bias. This study was also limited to middle school students in soccer teams. Due to differences in the growth process, it remains unclear whether the findings are applicable to other age groups, such as elementary or high school students. Although an association between hypoechoic findings and pain was observed, the cross-sectional design precluded determination of causality. Hypoechoic areas were defined as ≥ 2 mm based on a prior study [28]; however, evidence supporting this cutoff value is limited, and the optimal threshold remains unclear. Hypoechoic areas may reflect various underlying conditions, including ones that are reversible early and have specific pathological changes associated with pain. Further, all ultrasonographic measurements and image analyses were performed by a single examiner. Intra-rater reliability was assessed and found to be acceptable; however, inter-rater reliability was not evaluated, which may have introduced potential measurement bias. The number of painful knees was limited, resulting in a low events-per-variable ratio in the multivariable logistic regression analysis. Although clinically relevant variables were prespecified to reduce the risk of overfitting, the limited number of events may have affected the stability of the regression estimates. Finally, all participants were soccer players, which may have introduced selection bias and limited the generalizability of the results to athletes of other sports. Additionally, other factors that may influence the presence of hypoechoic changes and pain, such as training volume, level of sports participation, and type of sport, were not thoroughly investigated.

## Conclusions

This study demonstrated a significant association between patellar tendon-related pain and hypoechoic findings in middle school soccer players. The logistic regression analysis indicated that hypoechoic findings showed a stronger association with pain, compared with physical function measures. These findings suggest that US may provide useful information for identifying tendon characteristics observed in association with pain. However, due to the cross-sectional nature of this study, the causal relationship between hypoechoic findings and pain could not be determined. Further longitudinal research is warranted to elucidate the clinical significance of these morphological changes and their role in the development of patellar tendon-related disorders. In addition, these findings should be interpreted with caution because of the limited number of painful knees and the low events-per-variable ratio.

## Supporting information

S1 Table Participant-level sensitivity analysis. Participant-level sensitivity analysis comparing participant characteristics, physical function, and US findings between the painful and non-painful groups.
(DOCX)

S2 Data Anonymized knee-level dataset. Anonymized knee-level data underlying the analyses comparing painful and non-painful knees. (CSV)

S3 File STROBE checklist for the reporting of this observational study. (DOCX)
