# Supplementary material for: Association between patellar tendon-related pain and morphological abnormalities of the patellar tendon visualized using ultrasound imaging in middle school athletes
Source: PLoS One. 2026 Sep 8;21(9):e0357407. doi: 10.1371/journal.pone.0357407 (PMC13552770; doi:10.1371/journal.pone.0357407)
Supplement: Supplementary file 1 [file pone.0357407.s001.docx]

**Supplementary Table S1. Participant-level sensitivity analysis**

| **Variable** | **Pain (n=16)** | **No pain (n=99)** | **p-value** |
| --- | --- | --- | --- |
| Knee extension strength, kgf/kg^a^ | 0.6  (0.6-0.7) | 0.7  (0.6-0.8) | 0.22 |
| Hypoechoic-positive finding: n^b^ | 11  (69) | 18  (18) | <0.001 |
| Doppler-positive finding: n^b^ | 11  (69) | 28  (28) | 0.002 |

^a^: Analyzed using the Mann–Whitney *U* test. values presented as median (interquartile range: 25–75%)

^b^: Analyzed using the chi-square test. Values are presented as n (percentage).
